# Supplementary figures and images for: Leishmania major degrades murine CXCL1 – An immune evasion strategy
Source: PLoS Negl Trop Dis. 2019 Jul 1;13(7):e0007533. doi: 10.1371/journal.pntd.0007533 (PMC6625741; doi:10.1371/journal.pntd.0007533)

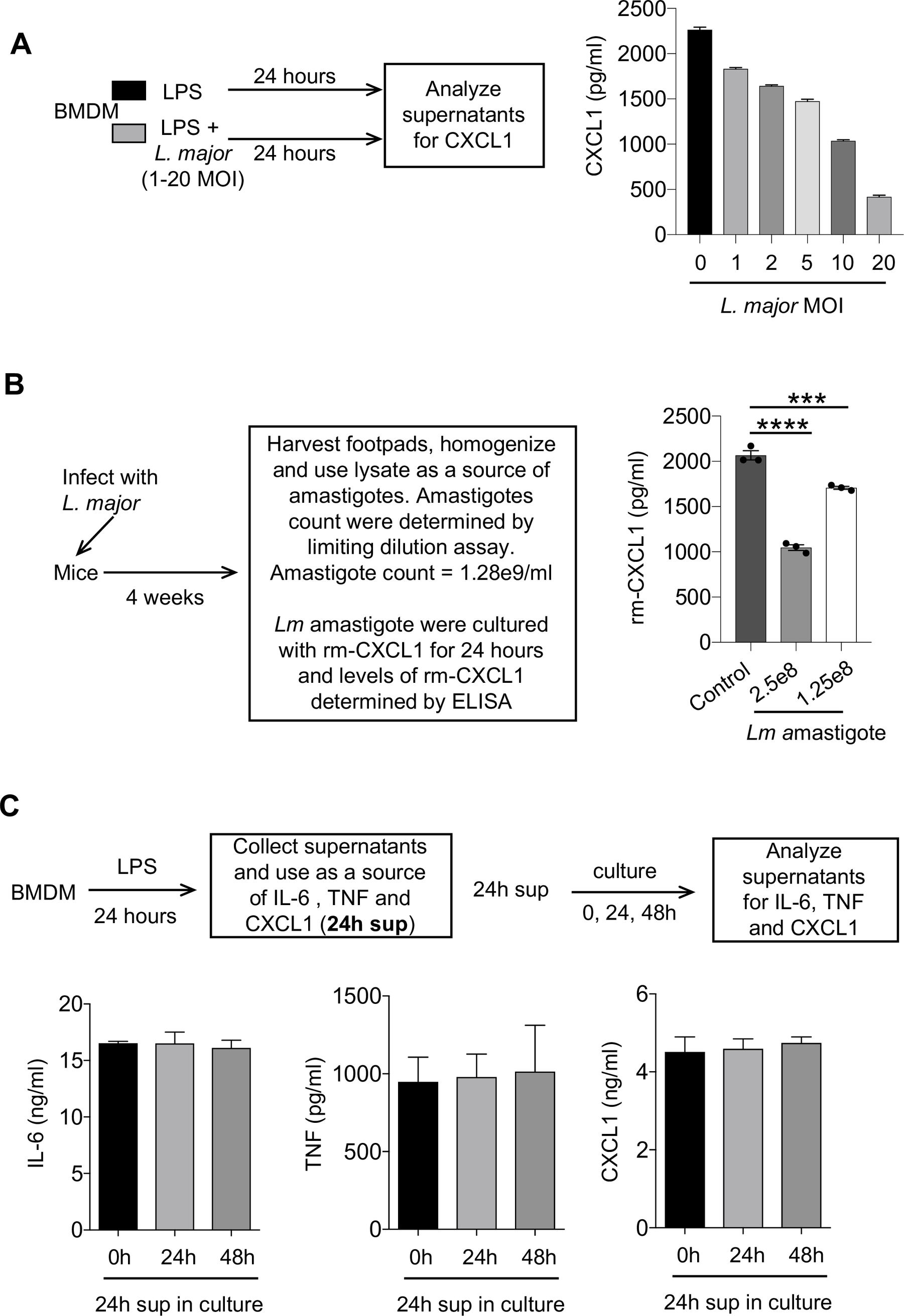

Supplement: S1 Fig — (A) BMDMs were treated with LPS (20 ng/ml) in the presence or absence of L. major (1, 2, 5, 10 and 20 MOI) for 24h and cell culture supernatants were analyzed for the indicated cytokines. (B) BALB/c mice were infected with 2 x 10^6^ L. major promastigotes / footpad, and 4 weeks later footpad harvested and homogenized. L. major burden in the footpad were determined by limiting dilution assay. Footpad lysates with L. major amastigote equivalent of 2.5e8 and 1.25e8 (based on limiting dilution assay) were cultured with rm-CXCL1 for 24 hours and the levels of rm-CXCL1 determined by ELISA. (C) Conditioned supernatants from 24h LPS (20 ng/ml) stimulated BMDMs were collected and further cultured for 0, 24, or 48h and stability of CXCL1, IL-6 and TNF in the culture were determined by ELISA. Data are representative of at least three independent experiments. Results are represented as mean ± SEM. ***P<0.001, ****P<0.0001. (TIF) [file pntd.0007533.s001.tif]

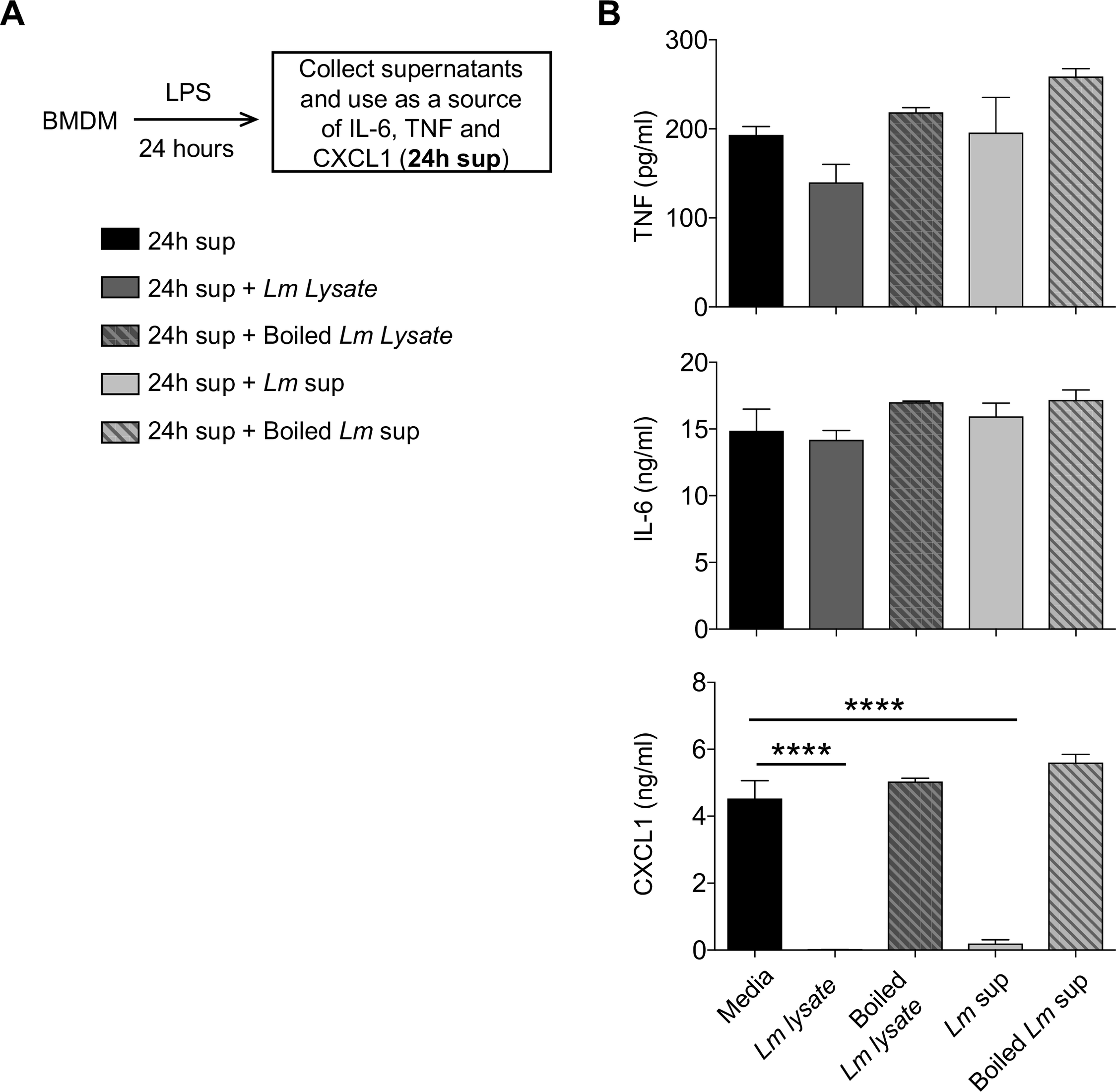

Supplement: S2 Fig — (A) Experimental Design. (B) Conditioned supernatants from 24h LPS (20 ng/ml) stimulated BMDMs were collected and subjected to 24h treatment with control or boiled (20 min at 100°C) Lm lysate or Lm sup. Levels of CXCL1, IL-6 and TNF were determined by ELISA. Results are represented as mean ± SEM. ****P<0.0001. (TIF) [file pntd.0007533.s002.tif]

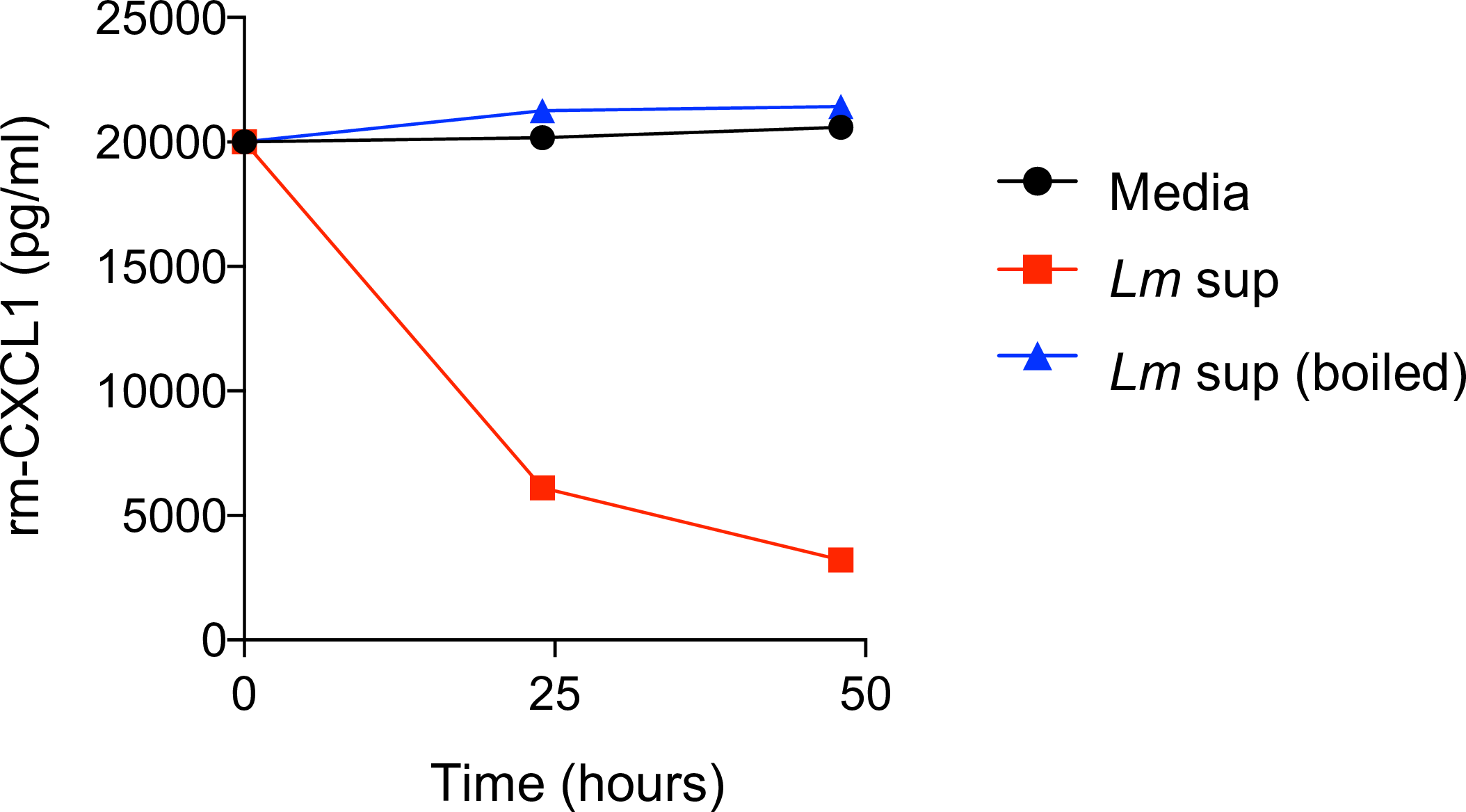

Supplement: S3 Fig — Rm-CXCL1 were left alone or treated with Lm sup (control or boiled) for up to 48 hours in culture. The quantity of rm-CXCL1 in the culture at 0, 24 and 48 h were determined by ELISA. Data are representative of at least three independent experiments. Results are represented as mean ± SEM. (TIF) [file pntd.0007533.s003.tif]

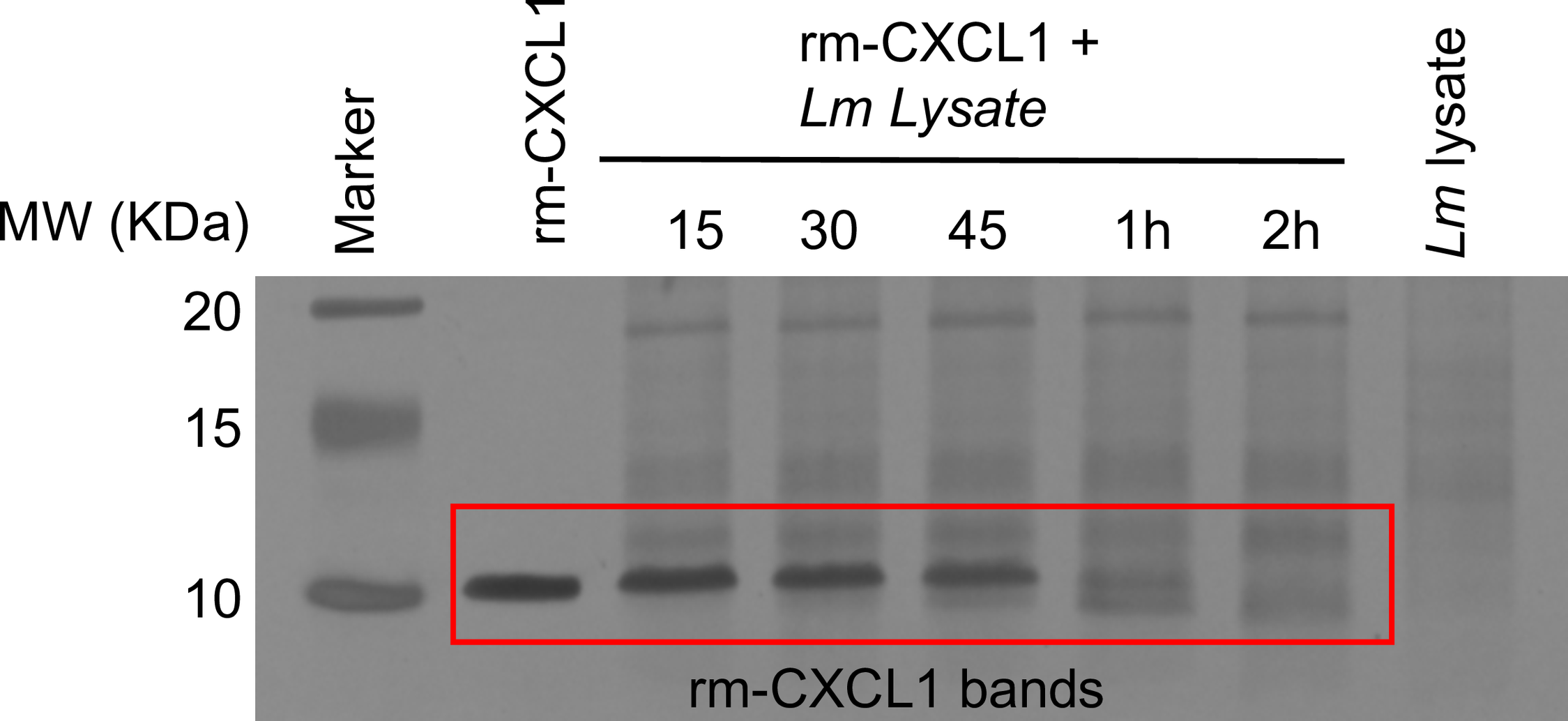

Supplement: S4 Fig — Rm-CXCL1 were left alone or treated with Lm lysate for acute time points as indicated to determine the precise time course of rm-CXCL1 cleavage. Silver staining was used to visualize rm-CXCL1. Data are representative of at least three independent experiments. (TIF) [file pntd.0007533.s004.tif]

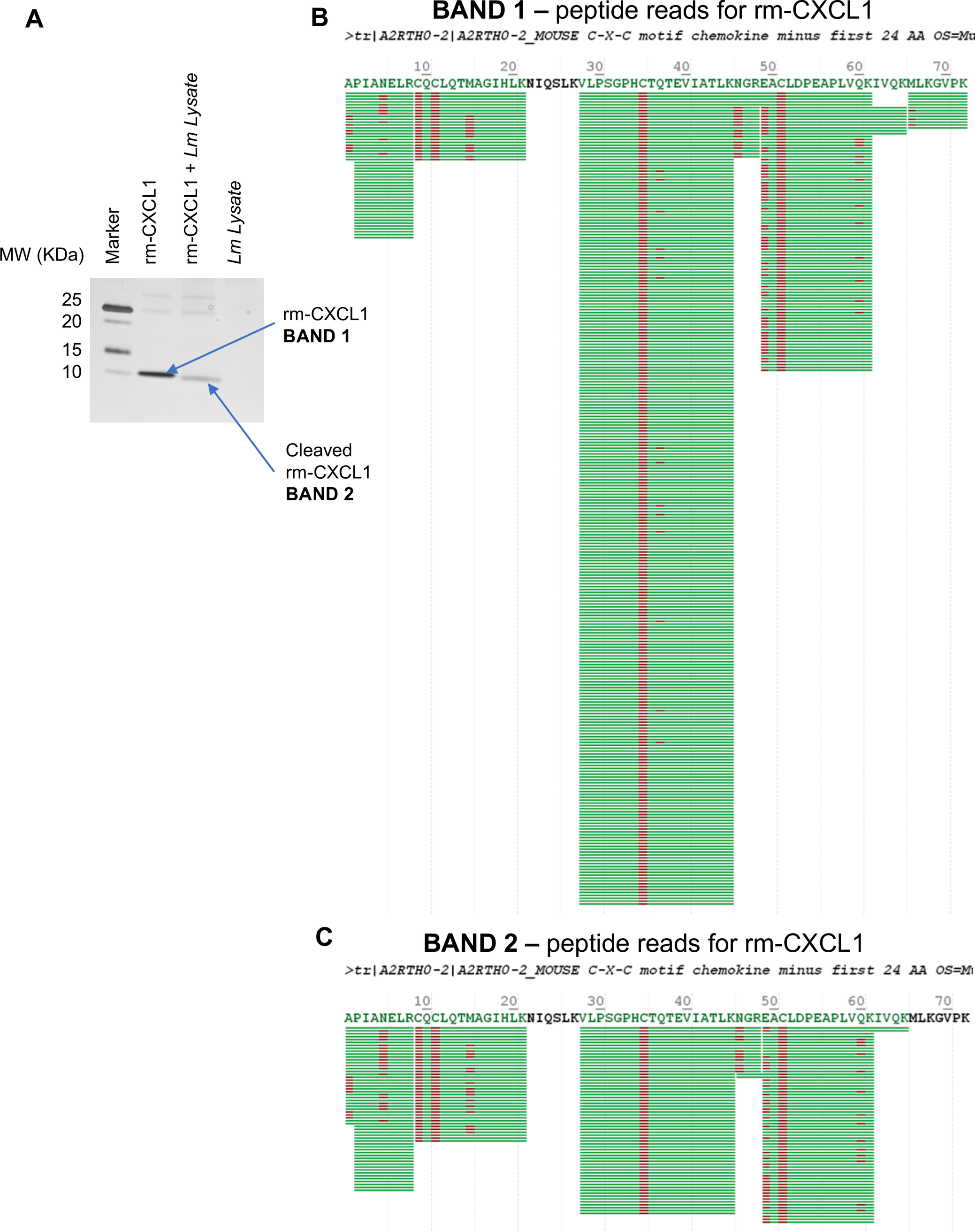

Supplement: S5 Fig — (A) A silver-stained SDS-PAGE showing cleavage of full length (BAND 1) and cleaved rm-CXCL1 (BAND 2). (B) Mass spectrometry analysis of BAND 1 (full length CXCL1) and tryptic peptide coverage analysis confirm a full length rm-CXCL1. (C) Mass spectrometry analysis of BAND 2 (cleaved CXCL1) and tryptic peptide coverage analysis confirm a C-terminal end cleavage after K65 residue. (TIF) [file pntd.0007533.s005.tif]

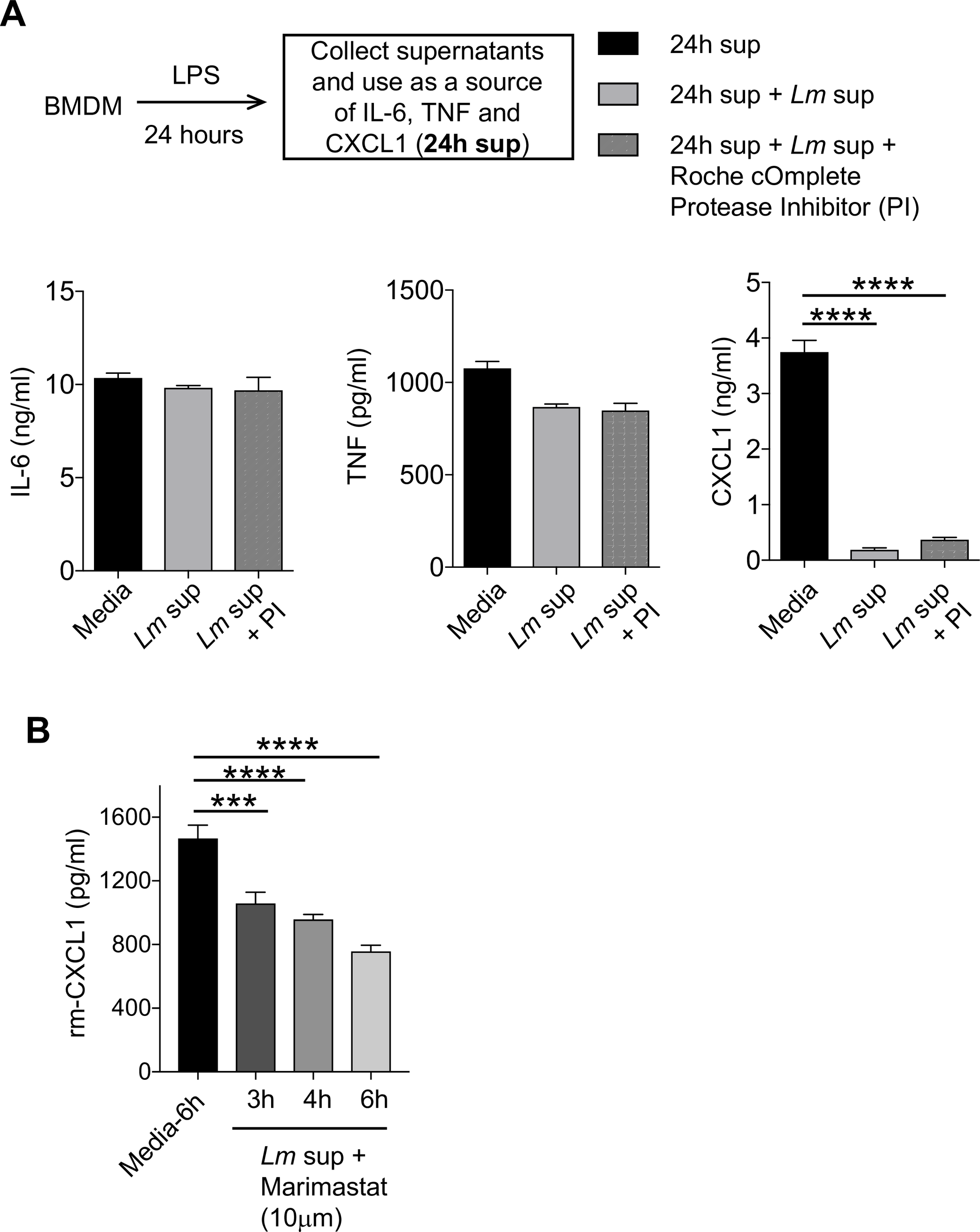

Supplement: S6 Fig — (A) Conditioned supernatants from 24h LPS (20 ng/ml)-stimulated BMDMs were collected and treated with Lm sup in the presence or absence of Roche cOmplete protease inhibitor. Levels of CXCL1, IL-6 and TNF were determined by ELISA. (B) Rm-CXCL1 was treated with Lm sup in the presence or absence of Marimastat (10μM) for indicated time-points and ELISA was performed to evaluate the levels of CXCL1. Data are representative of at least three independent experiments. Results are represented as mean ± SEM. ***P<0.001, ****P<0.0001. (TIF) [file pntd.0007533.s006.tif]
